# Gut Microbiota and Endometriosis: Exploring the Relationship and Therapeutic Implications

**DOI:** 10.3390/ph16121696

**Published:** 2023-12-07

**Authors:** Anjeza Xholli, Francesca Cremonini, Isabella Perugi, Ambrogio Pietro Londero, Angelo Cagnacci

**Affiliations:** 1 Academic Unit of Obstetrics and Gynecology, IRCCS Ospedale Policlinico San Martino, 16132 Genova, Italy; anj160583@yahoo.it (A.X.); francesca.cremonini96@gmail.com (F.C.); isabella.perugi@icloud.com (I.P.); 2 Department of Neuroscience, Rehabilitation, Ophthalmology, Genetics, Maternal and Infant Health, University of Genoa, 16132 Genova, Italy; ambrogiopietro.londero@unige.it; 3 Obstetrics and Gynecology Unit, IRCCS Istituto Giannina Gaslini, 16147 Genova, Italy

**Keywords:** endometriosis, gut microbiota, estrogen, dysbiosis, probiotics, leaky gut

## Abstract

Endometriosis is a common inflammatory disease affecting women of reproductive age, characterized by the growth of endometrial tissue beyond the uterus. In addition to gynecological manifestations, many endometriosis patients experience gastrointestinal symptoms, indicating a potential association between gut health and the disease. Recent studies have revealed alterations in the gut microbiota of individuals with endometriosis, including reduced diversity, microbial composition imbalances, and pathogenic bacteria. These changes can disrupt immune function, increase inflammation, and contribute to the chronic inflammatory state observed in endometriosis. Moreover, dysregulation of intestinal permeability may further exacerbate gastrointestinal symptoms in affected individuals. Understanding the role of the gut microbiota and intestinal permeability in endometriosis can provide valuable insights into disease pathogenesis, aid in non-invasive diagnostic approaches, and open new avenues for therapeutic interventions. Probiotics, in particular, have shown promise in improving endometriosis-associated pain symptoms and reducing endometriotic lesions in animal models. This review suggests that additional research and well-designed clinical trials are necessary to validate the potential diagnostic and therapeutic benefits of manipulating the gut microbiota in managing endometriosis and its gastrointestinal symptoms, thereby improving the quality of life for those affected.

## 1. Introduction

**Figure 1:**
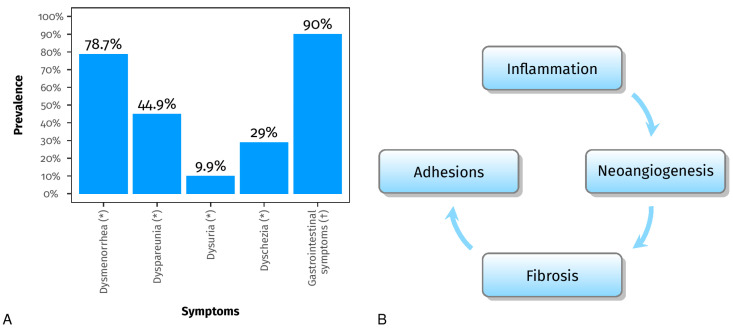
Panel (**A**) shows the prevalence of dysmenorrhea, dyspareunia, dysuria, and dyschezia (the 4-D’s) (*) [20] and the prevalence of gastrointestinal symptoms (†) [14]. Panel (**B**) shows the processes involved in endometriosis, which can disrupt anatomical structures and impair reproductive function [7,8,9,10,11].

Endometriosis is an inflammatory and estrogen-dependent condition, affecting approximately 6–10% of women in their reproductive years [1,2]. The pathogenesis and histological findings are characterized by endometrial glandular and stromal tissue growing beyond the uterus. It is classified into three types: superficial, ovarian, and deep endometriosis [3,4]. Although it is not considered a malignant disease, it can cause debilitating symptoms such as dysmenorrhea, dyspareunia, dysuria, and dyschezia (the 4-D’s) (Figure 1A) [5], and it may affect the fertility, psychological well-being, and social functioning of the patients. Moreover, it is one of the leading causes of access to the emergency department [6].

The growth of endometrial implants and the associated inflammation lead to increased production of pro-inflammatory cytokines [7,8,9,10]. These processes eventually lead to chronic inflammation, forming pelvic adhesions and endometriomas, which disrupt anatomical structures and impair reproductive function [9,11]. In addition to gynecological symptoms [12,13], up to 90% of patients with endometriosis experience gastrointestinal symptoms [14] (Figure 1A). Common gastrointestinal symptoms include bloating, nausea, constipation, diarrhea, and vomiting [14,15,16]. Recent advancements in genomics research and high-throughput sequencing technology have provided valuable insights into the relationship between human microbiota and female reproductive health [17,18]. It has been documented that the gut microbiota plays a significant role in various inflammatory conditions [19], and numerous studies have confirmed this association. 

The purpose of this review is to assess whether the available literature provides evidence of intestinal dysbiosis in endometriosis patients, potentially contributing to the gastrointestinal symptoms experienced by these patients. 

Additionally, the review aims to explore whether this condition could be alleviated through the consumption of probiotics, which might help restore a healthy balance of gut microbiota.

Another objective is to establish whether specific pathogens can serve as a distinctive “signature” of endometriosis once the presence of intestinal dysbiosis in these patients is established, offering a non-invasive diagnostic approach.

Lastly, the review explores the role of zonulin, a protein responsible for regulating intestinal permeability. The importance of this protein is becoming evident through numerous studies and could help elucidate the presence of gastrointestinal symptoms in endometriosis patients, even without direct intestinal involvement.

## 2. Methods

Given our project’s expansive and investigative nature, a narrative review was deemed the most suitable format for synthesizing the diverse range of literature sources available. As a comprehensive overview of the existing knowledge in the field, this approach enabled us to contextualize our research questions better and explore the various perspectives and interpretations of the subject matter. A comprehensive literature search was performed using PubMed, Scopus, and Google Scholar electronic databases. The keywords used included “endometriosis”, “gut microbiota”, “dysbiosis”, “probiotics”, and “leaky gut”. Only English articles published until 1 December 2023, were considered for this investigation. Subsequently, the authors assessed the findings, and this narrative review article summarizes the latest and pertinent data available. In addition, the evaluation of supplemental sources was carried out by taking into account the level of competence possessed by the authors, carefully examining the reference lists of the articles that were incorporated, and carrying out several different searches.

The publications underwent a screening process in which their titles and abstracts were evaluated to determine relevancy. References were organized using Zotero, a reference management tool (https://www.zotero.org/, 2 November 2023). Our search process mainly focused on systematic reviews, meta-analyses, and randomized controlled trials. The selection of papers was based on their pertinence and scholarly quality. The evaluation of scientific merit was established based on the publication of full-text articles in peer-reviewed academic publications. The determination of item relevance was guided by several criteria: pragmatism, which involved selecting the most valuable articles to provide a comprehensive overview, beginning with literature reviews; pluralism, which aimed to incorporate as many perspectives as possible; contestation, which involved examining conflicting data and engaging in argumentative debates; and consideration of publication date, with a preference for more recent publications. The exclusion criteria encompassed non-peer-reviewed articles or those that had been withdrawn. In the event of a discrepancy over the incorporated papers, a collaborative evaluation was conducted by three senior authors, resulting in a consensus being reached.

## 3. Mechanisms Underlying Endometriosis

According to Knapp (1999), the initial documentation of this pathological condition can be traced back to the late 17th century in Europe, specifically in the region encompassing the Netherlands and Flanders [21]. However, during the late nineteenth and twentieth centuries, the field of medicine started to investigate the etiological origins of diseases and describe their clinical manifestations. One of the prevailing etiological hypotheses is attributed to Sampson, who proposed his theory in 1927 in the United States. According to Sampson’s theory, endometriosis is believed to be caused by retrograde menstrual flow through the fallopian tubes, leading to the implantation of endometriotic cells on the peritoneum [22,23].

During the same period, Halban, a German colleague, developed a theory regarding the hematogenous and lymphatic dissemination of endometriosis [24,25]. This approach posits a potential rationale for the occurrence of endometriosis in distant anatomical sites and, concurrently, offers novel avenues for research by proposing the involvement of stem cells in the development of endometriosis implants [26,27,28,29]. Another notion proposed in the 1920s and credited to Meyer suggests that celomatic metaplasia may be responsible for the development of endometriosis. It is postulated that endometriosis occurs as a result of metaplastic changes in the celomatic epithelium [30,31]. The underlying rationale of this notion is rooted in the shared embryological derivation of perineal (or serous) cells and endometrial cells. Nevertheless, it is important to acknowledge that there are certain constraints to this idea. Specifically, metaplasia is contingent upon the advancement of time, leading us to anticipate a higher occurrence of endometriosis as individuals age. However, empirical evidence does not support this expectation. Furthermore, it is worth mentioning that the pleural serosa exhibits a shared embryonic origin, indicating a possible correlation with an increased prevalence of pleural endometriosis beyond what has been reported. Nevertheless, it is important to note that this idea was proposed in order to elucidate the etiology of deep endometriosis affecting the rectum-vaginal septum and the endometriomas [3,30].

The theory of induction, which builds upon the celomatic theory, is a proposed hypothesis aimed at elucidating the mechanisms underlying the development of endometriosis [32,33]. According to this idea, the development of endometriosis may be triggered by certain stimuli or substances present within the peritoneal cavity. The presence of these substances has the potential to induce the conversion of peritoneal cells into cells resembling those seen in the endometrium, resulting in the development of endometriotic lesions [32,33].

Furthermore, the etiology of endometriosis has been associated with other additional variables. The phenomenon of oxidative stress, characterized by an imbalance between reactive oxygen species (ROS) and antioxidants, has been proposed as a potential factor contributing to the pathophysiology of endometriosis [32,34]. Previous research has demonstrated that the presence of oxidative stress within the peritoneal cavity can induce inflammation, hence playing a significant role in the initiation and advancement of endometriotic diseases [32,35].

The potential role of a compromised immunological response in the etiology of endometriosis has also been suggested [23]. According to Seli et al., there is a belief that an impaired immune system may not effectively eliminate refluxed menstrual debris, hence enabling the establishment and proliferation of endometrial tissue outside the confines of the uterus [23].

Moreover, previous studies have suggested that the development of endometriosis may be influenced by genetic predisposition and hormonal variables [23,32]. Research findings indicate that there exists a positive correlation between the presence of endometriosis in women and a familial history of the disease, hence implying the involvement of genetic factors [32]. The development and progression of endometriosis have been linked to hormonal abnormalities, including elevated levels of estrogen [23].

None of the abovementioned arguments provide a comprehensive explanation for the occurrence of endometriotic tissue implantation in particular women, while others remain unaffected. This issue is particularly perplexing considering that retrograde menstruation has been reported in around 80% of women in their reproductive years, as documented by Kruitwagen et al. (1991) and Halme et al. (1984) [36,37]. Therefore, it is imperative to acquire a more comprehensive comprehension of the etiology of endometriosis in order to elucidate the underlying molecular pathways and, therefore, enhance the efficacy of therapeutic interventions. Guo emphasized that numerous therapy strategies, which are grounded in our existing molecular understanding of endometriosis, have failed to meet anticipated outcomes due to an incomplete comprehension of this complex disorder [38].

Endometriosis is a pathological condition characterized by hormonal dysregulation, and it has been observed that pharmacological interventions targeting this homeostatic imbalance demonstrate efficacy in managing the symptoms of this disease [39].

Nevertheless, a more comprehensive comprehension of the molecular pathways implicated in the formation and endurance of endometriotic tissue in locations beyond the uterus has the potential to pave the way for novel therapeutic interventions.

Individuals diagnosed with endometriosis commonly experience chronic pelvic pain, which is accompanied by an elevated presence of inflammatory constituents within the affected tissues [32,40]. Moreover, endometriosis is distinguished by an augmentation in the quantity of activated macrophages and pro-inflammatory cytokines [32,40].

Numerous studies have emphasized the correlation between elevated levels of pro-inflammatory cytokines and the occurrence of unpleasant sensations in women [41]. The generation of these compounds, which are linked to discomfort, may elucidate the reason why even small endometriosis implants can result in significant symptomatology (a discrepancy often exists between the severity of symptoms and the size of the endometriotic lesions).

## 4. The Bidirectional Relationship between Endometriosis and Microbiota

### 4.1. Gut Microbiota and Estrogens

Considering the immunological and hormonal alterations observed in individuals with endometriosis and the influence of the gut microbiota on immune and estrogen responses, there is a hypothesis that the gut microbiota plays a role in the development of endometriosis [19,42,43,44].

The gut microbiota can secrete enzymes such as β-glucuronidase and β-glucosidase, which can deconjugate estrogens and increase the reabsorption of free estrogen, leading to higher estrogen levels in the bloodstream [45,46,47,48,49,50]. 

The collection of genes encoding estrogen-metabolizing enzymes in the gut microbiota is commonly known as the “estrobolome” [51].

Analysis of the microbial genome has revealed that several bacterial genera in the gut microbiota, including Bacteroides, Bifidobacterium, *Escherichia coli*, and *Lactobacillus*, can produce β-glucuronidase [52].

Interestingly, research has shown a significant increase in *Escherichia coli* levels in the feces of endometriosis patients [53]. These findings suggest that the gut microbiota may contribute to elevated estrogen levels, creating an environment that promotes the progression of endometriosis [19,54].

Finally, in a study published in 2023, it seems that gut microbial β-glucuronidase (gmGUS) can become a potential biomarker for the early diagnosis of estrogen-induced diseases [55].

### 4.2. Dysbiosis and Chronic Inflammation

In 2002, an animal model study of female *Rhesus Macacus* with endometriosis showed a different microbiota composition than the healthy sample [56]. It appears that endometriosis was linked to reduced concentrations of *Lactobacilli* and elevated concentrations of Gram-negative bacteria. Furthermore, a higher incidence of intestinal inflammation was observed in monkeys with endometriosis compared to the healthy control group (the prevalence of intestinal inflammation in monkeys with or without endometriosis was determined through a retrospective analysis of necropsy reports).

The objective of the study by Svensson et al. [16] was to compare the gut microbiota of individuals with endometriosis and healthy controls. The researchers investigated whether there were variations in microbiota abundance within the endometriosis group based on disease localization, symptoms, or treatment. The study found a significant difference in overall gut microbia between healthy controls and individuals with endometriosis, with the gut microbiota of healthy controls showing higher diversity compared to patients with endometriosis. This finding suggests that there may be an association between reduced gut microbial diversity and the presence of endometriosis.

Imbalances in the composition of gut microbiota, known as dysbiosis, can disturb regular immune function, resulting in increased levels of proinflammatory cytokines, compromised immunosurveillance, and changes in immune cell profiles. These immune dysregulations are implicated in the chronic inflammatory state observed in endometriosis. The chronic inflammation associated with endometriosis creates an environment that promotes increased adhesions and angiogenesis, critical disease features. Adhesions can cause organs and tissues to stick together, leading to pain, functional impairment, and the development of new blood vessels that can provide oxygen and nutrients to the endometrial lesions, allowing them to grow and spread [11,57]. A study by Huang et al. [58], which compared the microbiome of peritoneal fluid, stool, and cervical mucus in endometriosis patients and healthy controls, observed an increased abundance of Gram-negative bacteria in the endometriosis group, including *Pseudomonas* and *Prevotella*, with the potential to release lipopolysaccharide (LPS). In the context of endometriosis, LPS can stimulate the activation of macrophages, leading to the production of inflammatory cytokines and chemokines. These inflammatory mediators can further promote the proliferation of endometriotic stromal cells, contributing to the growth and progression of endometriotic lesions [59].

### 4.3. Insights from Human and Animal Studies

The observed alterations in the microbiota of the gut, peritoneal fluid, and female reproductive tract in individuals with endometriosis compared to healthy controls have been well-documented in various human and animal studies, and at the same time, many experimental animal models have supported a bidirectional relationship between endometriosis and microbiota changes [11,16,58,60,61,62,63,64,65,66,67]. However, it is still not fully understood whether these alterations are a consequence of endometriosis or if they play a causative role in the development of the disease [68]. 

The findings of the main studies investigating the association between gut microbiota and endometriosis in animals and humans are summarized in Table 1 and Table 2, respectively. 

A study in mice with surgically induced endometriosis has shown that antibiotic treatment can reduce the size of endometriotic lesions, suggesting a potential role for the gut microbiota in disease progression. Additionally, fecal microbiota transfer from endometriotic mice to healthy mice resulted in the regrowth of lesions and associated inflammation [62]. Yuan et al. [66] conducted a prospective and randomized experiment using an animal model of endometriosis induced by intraperitoneal injection of endometrial tissues. Mice were divided into two groups: endometriosis and mock. The mice were sacrificed at four different time points to validate the model and collect fecal samples. 16S ribosomal-RNA gene sequencing was conducted to analyze the gut microbiota. The study found that the endometriosis and mock mice initially exhibited similar diversity and richness in their gut microbiota. However, after 42 days of modeling, distinct gut microbiota compositions were observed. Notably, mice with endometriosis showed an elevated Firmicutes/Bacteroidetes ratio, suggesting the induction of dysbiosis by endometriosis. 

In their human studies, Hernandes et al. [63] aimed to identify and compare bacterial patterns in endometriotic lesions, eutopic endometrium, and vaginal fluid from endometriosis patients to those found in the vaginal fluid and eutopic endometrium of control patients. Amplicon sequencing results showed similar microbial profiles in vaginal fluid, eutopic endometrium, and endometriotic lesions, with the most abundant genera being Lactobacillus, Gardnerella, Streptococcus, and Prevotella. No significant differences were observed in the diversity analysis of microbiome profiles between control and endometriotic patients. However, deep endometriotic lesions appeared to have a distinct bacterial composition, with a lower predominance of Lactobacillus and a higher abundance of Alishewanella, Enterococcus, and Pseudomonas. 

The study by Khan et al. (2021) [69] investigated the impact of antibiotic treatment, with or without GnRHa, on intrauterine infection and subsequent tissue changes in endometriosis. They collected endometrial/endometriotic samples from 53 women with endometriosis and 47 control women who received levofloxacin (LVFX) or GnRHa before surgery. The researchers used molecular and immunohistochemical analyses to assess the bacterial presence and various cellular markers. Results indicated that both LVFX and GnRHa + LVFX treatments significantly reduced specific bacterial genera in women with endometriosis. Specifically, in women with endometriosis, these treatments decreased Gardnerella, Prevotella, Acidibactor, Atopobium, Megasphaera, and Bradyrhizobium. Furthermore, the combination of GnRHa + LVFX demonstrated a greater reduction in chronic endometritis compared to GnRHa alone. These changes correlated with decreased macrophage infiltration (CD68), cell proliferation (Ki-67), and micro-vessel density (CD31) in endometria and endometriotic lesions, indicating an improved histological appearance of ovarian endometrioma. In conclusion, the study indicates that clinical administration of broad-spectrum antibiotics, with or without GnRHa, can be effective in reducing uterine infection and associated inflammation, cell proliferation, and angiogenesis in human endometriosis.

These results suggest that there is a dynamic interaction between the microbiota and endometriosis, where alterations in the microbiota can influence the development and progression of the disease, and conversely, the presence of endometriosis can affect the composition and function of the microbiota [70]. This dynamic relationship holds potential implications for non-invasive diagnostics and bacterial-based treatments in the future. 

**Table 1:** Animal studies that explored the bidirectional relationship between gut microbiota and endometriosis.

Study	N	Animal Species	Endometriosis Model	Sample	Technic	Aim and Conclusion
Bailey & Coe, 2002 [56]	18	Female Rhesus Monkeys (Macaca Mulatta)	Spontaneous occurring endometriosis, diagnosed with USG and MRI and confirmed by surgery and histology	Fecal samples	Coprocolture	***Aim:*** demonstrate that monkeys have an altered profile of intestinal microflora, in particular altered *Lactobacilli* ***Conclusion:*** decreased *Lactobacilli* and increased gram-negative anaerobes and facultative anaerobes
Yuan et al., 2018 [66]	Cases: 16 Controls: 20	C57BL6 mice	Induced: intraperitoneal injection of endometrial tissue from sacrificed donor mice	Fecal pellets	NGS Illumina: V4 16S rRNA	***Aim:*** determine the changes in gut microbiota in a murine endometriosis model by 16S ribosomal-RNA gene sequencing ***Conclusion:*** *Firmicutes/Bacteroidetes* ratio was elevated in mice with endometriosis, indicating that endometriosis may induce dysbiosis
Chadchan et al., 2019 [62]	Cases: 5 with endometriosis treated with vehicle and 4 with endometriosis and treated with broad spectrum antibiotics Controls: 5	C57BL/6 mice	Induced: autologous transplantation of endometrial tissue onto the peritoneal wall	Fecal pellets, peritoneal fluid, endometriotic lesions	NGS Illumina: V1-V9 16S rRNA	***Aim:*** investigate if altering gut microbiota with antibiotic treatment has any impact on endometriosis progression ***Conclusion:*** oral antibiotic treatment is effective in reducing the progression of endometriosis and administering fecal material from mice with endometriosis via oral gavage restored both the growth of endometriotic lesions and inflammation
Cao et al., 2020 [71]	Cases: 24 Controls: 8	Sprague Dawley rats	Induced: autologous transplantation of uterine tissue fragments onto the peritoneal wall	Fecal pellets	NGS Illumina: V3-V4 16S rRNA	***Aim:*** investigate if letrozole and SFZYD can act on microbiota, inhibiting the progression of lesions ***Conclusion:*** Letrozole and SFZYD reduce the inflammatory response in both ectopic and eutopic endometrial tissues, which could be associated with the decrease in the *Firmicutes*/*Bacteroidetes* ratio.
Ni et al., 2020 [72]	Cases: 16 Controls: 20	C57BL/6J mice	Induced: estrogen solution subcutaneous injection on days 1, 4, and 7, transplantation of endometrial fragments on day 8. Estrogen injection again on day 9, 11, and 14. After 3 weeks, mice were dissected to obtain faces from cecum.	Feces from cecum	NGS Illumina: V3-V4 16S rRNA	***Aim:*** uncover the interaction between fecal metabolomics and gut microbiota in mice with endometriosis ***Conclusion:*** The abnormal fecal metabolites, particularly those related to secondary bile acid biosynthesis and the alpha-linolenic acid pathways, influenced by dysbiosis, may serve as distinctive features in mice with endometriosis and as potential markers for distinguishing the disease

SFZYD, Shaofu Zhuyu decoction.

**Table 2:** Human clinical trials that explored the bidirectional relationship between gut microbiota and endometriosis.

Study	N	Age (Years)	Endometriosis Diagnosis Type	Sample	Technic	Aim and Conclusion
Khan et al., 2016 [73]	Cases: 32 Controls: 32 In each group 16 in treatment with GnRHa	21–52	Surgery and histology	Endometrial swabs and cystic fluid	NGS Illumina: 16S rRNA	***Aim:*** investigate microbial colonization in intrauterine environment and cystic fluid ***Conclusion:*** presence of sub-clinical infections in intrauterine environment and cystic fluid of ovarian endometriomas. Potential additional side effect of GnRHa treatment in promoting silent intrauterine and/or ovarian infections (abundance of *Streptococcaceae, Staphylococaceae, Enterobacteriaceae* and lowered *Lactobacillae* in GnRHa treated women)
Akiyama et al., 2019 [74]	Cases: 30 Controls: 39	20–44	Surgery and histology	Cervical Mucus	NGS Illumina: V5-V6 16S rRNA	***Aim:*** investigating pattern of microbiota in the cervical mucus ***Conclusion:*** *Enterobacteriaceae* and *Streptococcus* were more commonly found in women with endometriosis
Ata et al., 2019 [61]	Cases: 14 Controls: 14	18–45	Surgery and histology (only stage 3–4)	Stool sample Vaginal and endocervical swabs	NGS Illumina: V3- V4 16S rRNA	***Aim:*** comparing gut, vaginal, and cervical microbiota in endometriosis vs. controls ***Conclusion:*** no differences in species level analysis, but they found a possible difference at genus level analysis
Chen et al., 2020 [75]	Cases: 20 endometriosis, 19 adenomyosis and 7 adenomyosis-endometriosis Controls: 36	18–45	Surgery, histology, transvaginal ultrasound, and MRI	Cervical swabs and posterior fornix swabs	NGS Illumina: V3-V4 16S rRNA	***Aim:*** create a microbiota profile model for endometriosis and investigate and identify significant microbiota associated with endometriosis or adenomyosis conditions. ***Conclusion: *** Higher prevalence of *Atopobium* in endometriosis-adenomysosis group
Hernandes et al., 2020 [63]	Cases: 10 Controls: 11	18–50	Surgery and histology	Vaginal fluid, eutopic endometrium (collected by curettage), and endometrial lesion tissue samples (collected by surgery)	NGS Illumina: V3-V4 rRNA	***Aim:*** Investigate and compare the microbiome profile ***Conclusion: *** Vaginal fluid, eutopic endometrium, and endometriotic lesions: similar profiles in microorganisms but higher abundance of *Lactobacillus*, *Gardnerella*, *Streptococcus*, and *Prevotella* Deep endometriosis lesions: less lactobacillus and higher abundance of *Alishewanella*, *Enterococcus*, and *Pseudomonas*
Wei et al., 2020 [65]	Cases: 36 Controls: 14	23–44	Surgery and histology	Lower reproductive tract swabs: lower third of the vagina, posterior vaginal fornix, cervical mucus Upper reproductive tract samples: surgery (endometrial and peritoneal lavage)	NGS Ion Torrent PGM: V4-V5 16S rRNA	***Aim:*** study the flora distribution and bacterial community across the upper and lower reproductive tract ***Conclusion:*** shift in microbiota distribution starting from the cervical samples (microbiota in cervical samples as an indicator for the risk of endometriosis) and progressively increasing upward the reproductive tract, decreased *Lactobacillus* in lower tract, enriched *Sphingobium* sp. and *Pseudomonas viridiflava* in endometrium and peritoneal fluid
Khan et al., 2021 [69]	Cases: 53 (21 untreated, 11 GnRHa, 15 LVFX, 6 LVFX+ GnRHa) Controls: 47 (11 untreated, 12 GnRHa, 10 LVFX, 14 LVFX+ GnRHa	18–51	Surgery and histology	Endometrial samples	NGS Illumina: V5-V6 16S rRNA Immunohistochimical analysis: Ab against CD138, CD68, Ki-67, and CD31	***Aim:*** demonstrate the hypothesis that antibiotic treatment with or without GnRHa may decrease intrauterine infection with consequent decrease in tissue inflammation, cell proliferation and angiogenesis in human endometriosis ***Conclusion:*** Decreased *Gardnerella*, *Prevotella, Acidibactor*, *Atopobium, Megasphaera*, and *Bradyrhizobium* in patients with endometriosis in treatment with LVFX or LVFX + GnRHa, reduced occurrence rate of chronic endometritis after GnRHa + LVFX treatment comparison to GnRHa treatment group and decreased CD68, Ki-67, and CD31

LVFX, levofloxacin; GnRHa, gonadotropin-releasing hormone analogues.

## 5. Microbiome Testing: A Promising Non-Invasive Approach for Endometriosis Diagnosis

**Figure 2:**
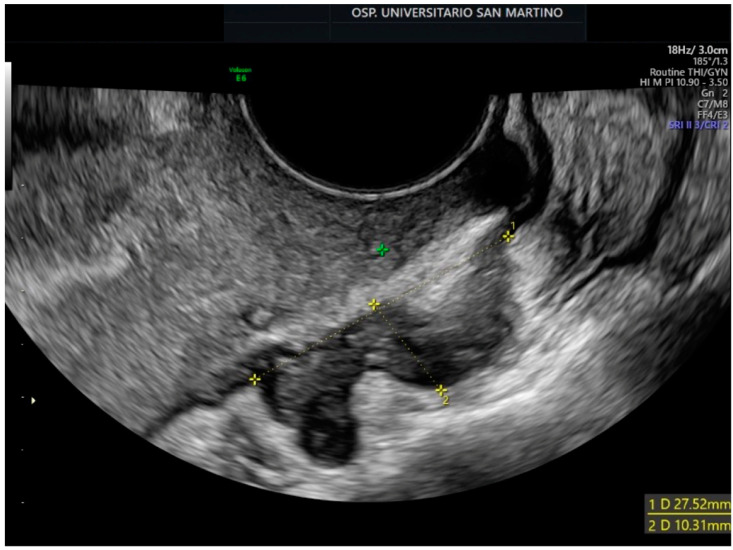
The figure displays the presence of an endometriosis implant on the rectum in a patient undergoing a transvaginal ultrasound at our San Martino Hospital in Genoa. This patient experienced symptoms including dyschezia, rectal tenesmus, alternating episodes of constipation and diarrhea, as well as painful intestinal spasms. The yellow calipers represent the measurements. The green one represents the ultrasound machine’s pointer.

The potential use of microbiome testing as a non-invasive method for detecting endometriosis is promising. The average delay in diagnosing endometriosis, due to the nonspecific nature of its symptoms [76], is significant, ranging from 4 to 12 years, and laparoscopic diagnosis, the diagnostic gold standard, is an invasive procedure. Improved imaging techniques such as transvaginal ultrasound (Figure 2) and MRI have enhanced diagnostic accuracy for deep endometriosis. However, the availability of skilled professionals and the limitations of diagnosing superficial endometriosis remain. In the future, a simple microbiota test could complement existing imaging modalities, offering a non-invasive approach to diagnosing endometriosis.

To facilitate a non-invasive diagnosis of endometriosis via microbiome testing, it is necessary to classify the microorganisms commonly found in endometriosis patients’ microbiomes and identify differences between their microbiota and that of the healthy population [77,78,79].

A study by Khan et al. [80] provides interesting findings: according to their research, there was a significant increase in the levels of *Escherichia coli* (*E. coli*) in the menstrual blood of women with ovarian endometriomas and superficial peritoneal lesions compared to women with ovarian endometriomas alone.

Instead, Huang et al. [58] enrolled 41 women, including 20 healthy controls and 21 with endometriosis. The researchers collected samples from three body sites: feces, cervical mucus, and peritoneal fluid to investigate the microbiome composition in endometriosis patients. The analysis revealed significant differences in microbial composition among the three sites, and the results indicated that endometriosis patients had distinct microbial communities compared to the control group, particularly in the feces and peritoneal fluid. 

The peritoneal fluid of endometriosis patients showed an increased abundance of pathogens, while the feces exhibited a depletion of protective microbes. The genera Ruminococcus and Pseudomonas were identified as potential biomarkers in the gut and peritoneal fluid, respectively. Additionally, the researchers developed novel classifiers for endometriosis based on selected taxa using a robust machine-learning method. By employing machine learning techniques, the researchers could analyze the samples’ microbial composition and identify taxa patterns that were most indicative of endometriosis. The results suggested that the gut microbiota may have a higher diagnostic value for endometriosis than the cervical microbiota [58]. Further research and validation studies are necessary to confirm these microbial classifiers’ accuracy, reliability, and application in clinical settings.

## 6. Restoring Gut Microbiota Balance in Endometriosis: The Potential of Probiotics as a Therapeutic Approach

Correcting gut microbiota imbalances to restore their normal functional state has emerged as a potential therapeutic approach for various diseases, including endometriosis [81,82]. The regulation of gut microbiota can be achieved through several strategies, such as using antibiotics, fecal bacteria transplantation, probiotics, and dietary interventions with specific nutrients [83,84,85,86].

In particular, probiotics are live microorganisms that confer benefits when administered in adequate amounts by promoting a healthy gut microbiota. Indeed, recognizing an endometriotic signature in the intestinal microbiota could open up new possibilities for therapeutic approaches using probiotics and prebiotics as a non-surgical treatment option for endometriosis.

A study investigated the effects of supplementing Akkermansia [87], identified as a promising therapeutic probiotic for addressing metabolic disorders in the context of gynecological disorders, especially those susceptible to inflammatory bowel disease (IBD) like endometriosis. The study concluded that a careful evaluation is necessary, and further studies are essential to clarify its application areas and establish the safety of Akkermansia.

In another study, the impact of Lactobacillus acidophilus on peripheral blood mononuclear cells (PBMCs) in women with endometriosis was investigated [88]. Endometriosis patients displayed increased production of pro-inflammatory cytokines IL-1 and IL-6 by PBMCs compared to those without endometriosis. *L. acidophilus* supplementation further heightened cytokine levels; however, after 48 h, bacterial cells exhibited modulatory properties, leading to a reduction in cytokine production. The findings suggest that probiotics, particularly *L. acidophilus*, might have potential therapeutic benefits for endometriosis.

In a randomized pilot placebo-controlled trial [89] involving women diagnosed with stage 3 and 4 endometriosis, LactoFem^®^ (a formulation containing Lactobacillus) was administered orally once a day for 8 weeks. The study included 37 participants without hormonal treatment in the last three months. The participants were assessed for pain severity using Visual Analogue Scale (VAS) scores for dysmenorrhea, dyspareunia, and chronic pelvic pain at baseline, 8 weeks, and 12 weeks post-intervention. The study showed that Lactobacilli had beneficial effects on endometriosis-associated pain, specifically dysmenorrhea, and chronic pelvic pain. The most significant improvement in dysmenorrhea was observed after 8 weeks of Lactobacilli consumption. In addition, Lactobacilli significantly reduced overall discomfort during the study. These findings suggest that the administration of Lactobacilli may positively impact endometriosis-related pain symptoms. However, it is important to note that this was a pilot study with a relatively small sample size, and further randomized trials with larger participant groups are needed to better evaluate the effectiveness and long-term effects of lactobacilli in endometriosis management.

Studies in mice have demonstrated that oral administration of Lactobacillus can reduce endometriotic lesions. This effect is thought to be mediated through increased IL-6 and IL-12 concentrations and enhanced natural killer (NK) cell activity [90,91,92,93]. In endometriosis, dysbiosis and inflammation can lead to impaired NK cell activity, and the administration of Lactobacillus probiotics has been shown to reverse this immune dysregulation. Notably, Lactobacillus probiotic treatment not only improved existing endometriosis but also showed the capability of preventing the growth of endometriotic lesions in rats [91].

These findings highlight the potential therapeutic benefits of Lactobacillus probiotics in managing endometriosis [11,19].

## 7. Role of Intestinal Permeability in Endometriosis-Related Gastrointestinal Symptoms

The association between dysbiosis and increased intestinal permeability, commonly referred to as “leaky gut”, has been demonstrated. This heightened permeability allows toxins, bacteria, and other harmful substances to enter the bloodstream, triggering an immune response and consequently leading to a chronic inflammatory state that could contribute to the progression of endometriosis [94,95].

Mohling et al. [94] conducted a pilot project at the University of Tennessee that aimed to investigate whether patients with laparoscopically confirmed endometriosis exhibit higher rates of impaired intestinal permeability compared to healthy controls and pelvic pain patients without endometriosis. The study utilized a lactulose/mannitol oral challenge to assess intestinal permeability. Out of 20 patients with laparoscopically defined endometriosis, 45% had impaired intestinal permeability, whereas none of the 9 patients without endometriosis (control subjects) showed impairment (*p* = 0.027). The study suggests a potential association between impaired intestinal permeability and endometriosis, emphasizing the need for further research to understand its role in the pathogenesis and potential diagnostic implications for endometriosis.

In 2000, a novel protein called zonulin was discovered. It is considered an essential mediator in controlling the permeability of intestinal epithelial cells by modulating tight junction integrity. Its dysregulation or overexpression may contribute to various pathological conditions, including inflammatory bowel diseases, celiac disease, and other disorders characterized by abnormal intestinal permeability. Moreover, a notable association was observed between zonulin levels and the outcomes of the lactulose/mannitol oral challenge, a test employed to assess intestinal permeability [96].

**Figure 3:**
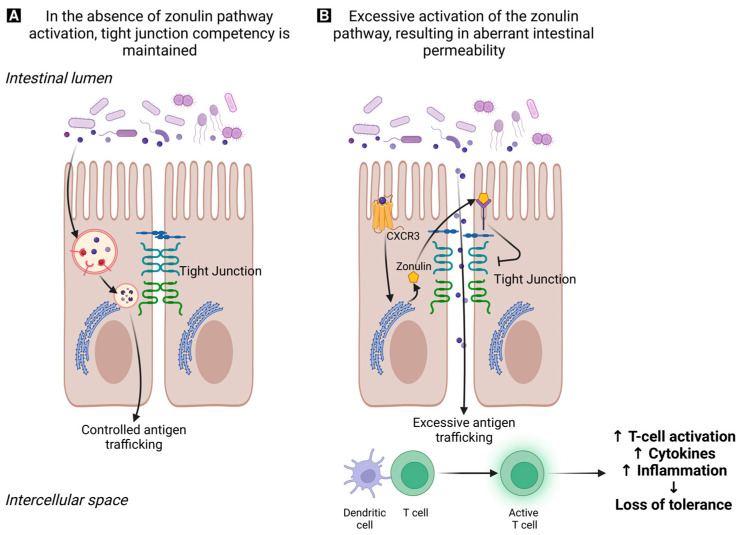
This illustration depicts the role of zonulin in regulating intestinal permeability. Panel (**A**) illustrates the circumstances when a stable gut microbiota is present. The processes of transcellular antigen sampling by enterocytes and antigen sampling by luminal dendritic cells play a crucial role in regulating antigen trafficking between the intestinal lumen and the submucosa. This process ultimately determines whether the body develops tolerance or immunity toward non-self-antigens. Panel (**B**) depicts the conditions of gut dysbiosis that result in zonulin release, causing a transient opening of tight junctions. Chronic gut dysbiosis results in a shift from regulated antigen transportation to an elevated antigen transportation process dependent on zonulin due to alterations in gene expression associated with tight junctions. The heightened transportation of antigens results in an inflammatory response, which in turn augments the permeability of the gastrointestinal tract. This process leads to the production of pro-inflammatory cytokines, activation of T-cells, and a breakdown of immune tolerance in people with impairments in immunoregulation. Illustration created with BioRender.com.

Excessive zonulin release weakens the intestinal barrier, causing increased antigen leakage through tight junctions. Normal tight junctions open briefly in response to specific triggers; however, in an imbalanced state, prolonged zonulin-triggered opening causes an excessive passage of antigens through the intestinal barrier (Figure 3) [97].

This heightened antigen movement can cause inflammation and improper activation of the immune response, breaking tolerance to abnormal self-antigens. This process can trigger autoimmune reactions in genetically predisposed individuals.

Gut dysbiosis induces zonulin release, temporarily opening tight junctions. With persistent dysbiosis, controlled antigen movement shifts to zonulin-dependent trafficking, influenced by tight junction gene expression changes. This heightened antigen movement prompts further inflammation, intensifying gut permeability and fostering pro-inflammatory cytokine production. The resulting inflammatory condition in the intestine may provide an explanation for the gastrointestinal symptoms observed in patients with endometriosis [97,98].

As highlighted by Maroun et al. [14], among women with histologically confirmed endometriosis, 90% reported experiencing gastrointestinal symptoms. Bloating was the most commonly reported symptom, affecting 83% of women. Interestingly, only a small percentage of women with gastrointestinal symptoms had bowel endometriosis, suggesting that gastrointestinal symptoms in endometriosis are not solely dependent on bowel involvement. The presence of gastrointestinal symptoms in women with endometriosis, even without bowel involvement, raises the possibility of misdiagnosis or overlap with conditions such as irritable bowel syndrome (IBS) or inflammatory bowel disease (IBD). The similarity in symptoms between endometriosis and IBS/IBD can make it challenging to differentiate between the two conditions [99,100,101]. 

Future research could explore the potential alteration of intestinal permeability in endometriosis patients with severe gastrointestinal symptoms. Zonulin could be investigated as a marker of intestinal permeability in these patients. Assessing the levels of zonulin and examining the integrity of the intestinal barrier could provide insights into the relationship between endometriosis and gastrointestinal symptoms.

Additionally, investigating the effects of probiotic administration in endometriosis patients with gastrointestinal symptoms could provide valuable information on the potential benefits of probiotics in improving symptoms and quality of life.

## 8. Conclusions

Conducting well-designed clinical trials to evaluate the efficacy of specific probiotic strains, dosages, and treatment durations could help determine the potential benefits of probiotic interventions in managing gastrointestinal symptoms associated with endometriosis. In addition, exploring the role of intestinal permeability in endometriosis and investigating the effects of probiotics on gut health can provide valuable insights into the underlying mechanisms of the gastrointestinal symptoms of endometriosis. While there is currently no empirical evidence to support elevated zonulin levels in endometriosis, its relationship with increased intestinal permeability implies a possible link. Our findings highlight the importance of additional research to establish a causal relationship. The hypothesis of zonulin in endometriosis is still being debated scientifically, with additional data needed for validation or rejection. This issue is emphasized in our manuscript. Our idea is notable for its original combination of zonulin’s role in intestinal permeability with endometriosis, which opens up new research possibilities. Probiotics may help modulate the gut microbiota, strengthen the intestinal barrier, and reduce inflammation, which could alleviate gastrointestinal symptoms. By better understanding the gut-related aspects of endometriosis and the potential effects of probiotics, we can explore new avenues for managing gastrointestinal symptoms and improving the quality of life for individuals with endometriosis. However, further research is needed to establish the optimal probiotic interventions and their specific mechanisms of action in the context of endometriosis.

## Data Availability

Data sharing is not applicable.
